# Clofarabine Improves Relapse-Free Survival of Acute Myeloid Leukemia in Younger Adults with Micro-Complex Karyotype

**DOI:** 10.3390/cancers12010088

**Published:** 2019-12-30

**Authors:** Laurène Fenwarth, Nicolas Duployez, Xavier Thomas, Nicolas Boissel, Sandrine Geffroy, Alice Marceau-Renaut, Denis Caillot, Emmanuel Raffoux, Emilie Lemasle, Jean-Pierre Marolleau, Céline Berthon, Meyling H. Cheok, Pauline Peyrouze, Arnaud Pigneux, Norbert Vey, Karine Celli-Lebras, Christine Terré, Claude Preudhomme, Hervé Dombret

**Affiliations:** 1 Laboratory of Hematology, CHU Lille, 59000 Lille, France; laurene.fenwarth@chru-lille.fr (L.F.); nicolas.duployez@chru-lille.fr (N.D.); sandrine.geffroy@chru-lille.fr (S.G.); alice.marceau@chru-lille.fr (A.M.-R.); 2 Jean-Pierre AUBERT Research Center, University Lille, Inserm, UMR-S 1277, 59000 Lille, France; meyling.cheok@inserm.fr (M.H.C.);; 3 Lyon Sud, University Hospital, 69495 Pierre-Bénite Lyon, France; xavier.thomas@chu-lyon.fr; 4 ALFA Group, 75010 Paris, France; karine.celli-lebras@aphp.fr (K.C.-L.); herve.dombret@aphp.fr (H.D.); 5 Hematology Department, Saint-Louis Hospital, 75010 Paris, France; nicolas.boissel@aphp.fr (N.B.); emmanuel.raffoux@aphp.fr (E.R.); 6 EA3518, Saint-Louis Institute for Research, Paris University, 75010 Paris, France; 7 Hematology Department, Dijon University Hospital, 21000 Dijon, France; denis.caillot@chu-dijon.fr; 8 Hematology Department, Henri Becquerel Cancer Center, 76038 Rouen, France; emilie.lemasle@chb.unicancer.fr; 9 Hematology Department, Amiens University Hospital, 80054 Amiens, France; marolleau.jean-pierre@chu-amiens.fr; 10 Hematology Department, CHU Lille, 59000 Lille, France; celine.berthon@chru-lille.fr; 11 Hematology Department, Bordeaux Haut-Leveque University Hospital, 33600 Pessac, France; arnaud.pigneux@chu-bordeaux.fr; 12 Onco-Hematology Department, Paoli-Calmettes Cancer Institute, 13009 Marseille, France; veyn@unicancer.ipc.fr; 13 Laboratory of Hematology, André Mignot Hospital, 78157 Le Chesnay, France; cterre@ch-versailles.fr

**Keywords:** acute myeloid leukemia, snp-array, micro-complex karyotype

## Abstract

Acute myeloid leukemia (AML) encompasses heterogeneous entities with dismal outcomes. Intermediate and unfavorable-risk AML represent the most difficult-to-treat entities. We recently reported the benefit of the clofarabine-based consolidation (CLARA) regimen compared to the standard high-dose cytarabine (HDAC) regimen in younger AML patients. Here, we aimed at assessing the clinical significance of single-nucleotide polymorphism (SNP)-array alterations and their interactions with chemotherapy regimens. A SNP-array was successfully performed in 187 out of the 221 intent-to-treat patients (CLARA arm: *n* = 92 patients, HDAC arm: *n* = 95 patients). The CLARA regimen did not significantly improve relapse-free survival (RFS) among patients who displayed a complex karyotype when compared to the HDAC regimen (4-year RFS (4y-RFS): 36.4% vs. 18.8%, respectively; *p* = 0.134). Defining micro-complex karyotypes from at least four SNP-array lesions enabled us to refine and enlarge the subset of adverse patients. In such patients, the CLARA regimen significantly improved RFS compared to the HDAC regimen (4y-RFS: 44.4% vs. 13.8%, respectively; *p* = 0.004). From our study cohort, 8% of patients displayed *TP53* mutations, which were associated with an impaired RFS (4y-RFS: 20.0% vs 43.7%; *p* = 0.029). In a multivariate analysis, micro-complex karyotypes remained the sole poor prognostic factor in the HDAC arm (hazard ratio (HR) = 2.324 (95% confidence interval (CI) = 1.337–4.041), *p* = 0.003). The SNP array represents a powerful and reproductive approach to refine adverse AML patients that may benefit from alternative consolidation regimens.

## 1. Introduction

Acute myeloid leukemia (AML) encompasses heterogeneous entities, but its global outcome remains of concern. Recurrent numerical and structural cytogenetic abnormalities are among the most important prognostic factors in AML and are now routinely used to inform disease classification and stratify patients [1]. Among AML cytogenetic subgroups, complex karyotypes stand as one of the most difficult-to-treat. On the other hand, nearly 50% of AML patients show a normal karyotype in conventional cytogenetics, and about 25% have uncommon aberrations [2]. In this context, high resolution approaches such as single-nucleotide polymorphism (SNP)-array karyotyping are attractive to better characterize unrecognized or cryptic copy number alterations (CNAs) and copy-neutral-loss of heterozygosity (CN-LOH), and these approaches may provide new information in AML diagnosis and prognostication [3]. Some studies have shown the feasibility of interrogating AML genomes with such technologies [4,5]. They have led to the discovery of novel recurrent gene deletion, amplification, and CN-LOH encompassing important targets including *TET2*, *CBL*, *EZH2* or *FLT3* (reviewed in [6]). However, few studies have focused on the prognostic impact of SNP-array lesions in clinical practice. Recently, we reported the benefits of the clofarabine combination with intermediate-dose cytarabine (CLARA arm) compared to standard high-dose cytarabine (HDAC arm) as consolidation chemotherapy in AML patients with intermediate or unfavorable cytogenetics who were enrolled in the randomized Acute Leukemia French Association (ALFA)-0702 trial and the subsequent CLARA study [7]. Here, we describe the SNP-array profiling of this well-annotated cohort of AML patients and evaluate the prognostic impact of these genetic alterations on clinical outcomes and their interactions with chemotherapy regimens.

## 2. Methods

### 2.1. Patients and Treatment

From March 2009 to September 2013, 713 patients aged 18–59 years old with previously untreated de novo AML were included in the phase II randomized multicenter ALFA-0702 trial (ClinicalTrials.gov identifier: NCT00932412) [7]. Informed consent for both treatment and genetic analysis was obtained before the inclusion in accordance with the Declaration of Helsinki. The study protocol was approved in December 2008 by the Institutional Review Board of the French Regulatory Agency and the Ethics Committee Sud-Est IV, France (ID: 08/099). Patients with intermediate or unfavorable-risk AML who had achieved first complete remission (CR) after one or two induction courses and who had no identified human leukocyte antigen-compatible donor were randomly assigned to the CLARA study (*n* = 221 patients). The CLARA study compared three clofarabine-based consolidation courses (CLARA arm) to three high-dose cytarabine courses (HDAC arm). Among the 221-intent-to-treat patients, the SNP-array was performed in 187 (85%) who had available genomic DNA.

### 2.2. SNP-A Karyotyping

Bone marrow or peripheral blood samples at diagnosis were analyzed by a Cytoscan HD array (ThermoFisher (Santa Clara, CA, USA)) according to the manufacturer’s instructions, as previously described [8]. Data were screened according to the Chromosome Analysis Suite (ChAS) 3.1 software (ThermoFisher), with stringent filters. In order to exclude germline alterations, only CNAs larger than 20 kb—including at least 20 consecutive markers and telomeric and interstitial copy-neutral-loss of heterozygosity (CN-LOH) over 3 and 10 Mb, respectively—were considered for this analysis. Additionally, all CNAs and CN-LOH fulfilling these criteria were approved through a visual inspection, and they were annotated based on the human genome version 19 (hg19) of the University of California Santa Cruz (UCSC) Genome Browser.

### 2.3. Mutational Analysis

The genomic data of the *NPM1*, *CEBPA* and *FLT3*-ITD mutations were provided by standard procedures, as previously described [3,9]. The *TP53* mutations were screened by high-throughput sequencing (full gene). Briefly, libraries were prepared by using the Haloplex System (Agilent Technologies, Santa Clara, CA, USA) according to the manufacturer’s instructions, and then they were run on a MiSeQ (Illumina, San Diego, CA, USA). Raw data were processed with the SureCall (Agilent Technologies, Santa Clara, CA, USA) and SeqNext (JSI Medical systems Corp., New York, NY, USA) softwares.

### 2.4. Statistical Analysis

The principal endpoint was relapse-free survival (RFS). RFS was defined from the date of the CR/CR with incomplete platelet recovery (CRp) to the date of relapse or death or last contact for patients alive in continuous CR/CRp. RFS was estimated by the Kaplan–Meier method and compared by the log-rank test. Comparisons were performed by the Wilcoxon rank-sum test for continuous variables and by χ^2^ or the Fisher exact test for categorical variables. The hazard ratio (HR) was given with a 95% confidence interval (95% CI). Multivariate analyses were performed by using the Cox proportional hazard model. Statistical analyses were carried out with the use of the SPSS Statistics V22.0 software (IBM Corp., Chicago, IL, USA). All *p*-values were two-sided, with *p* < 0.05 considered as statistically significant.

## 3. Results and Discussion

The patient characteristics are summarized in Table S1. The SNP-array was performed in 187 randomized patients (CLARA arm, *n* = 92; HDAC arm, *n* = 95). A total of 801 SNP-array alterations were identified. The median number of abnormalities was two per patient (first quartile: 1; third quartile: 4; range: 0–46) and did not significantly differ between both randomization arms (*p* = 0.41) (Table S1). CNAs accounted for the most common lesions (losses: 54%, gains: 38%; vs. CN-LOH: 8%). The third quartile (75th percentile) was found to be the optimal cut-off to discriminate AML patients into prognostic subgroups. Consequently, AML genomes harboring four or more SNP-array lesions (including CNA and CN-LOH) were defined as the “micro-complex” karyotype.

Overall, a total of 27 patients displayed the complex karyotype (i.e., at least three chromosomal abnormalities) in conventional cytogenetics, while 56 patients harbored the “micro-complex” karyotype, including 20 patients with the complex karyotype. The micro-complex karyotype was not found in the seven remaining patients, possibly due to low bone marrow/blood blast infiltration and/or balanced alterations.

**Figure 1:**
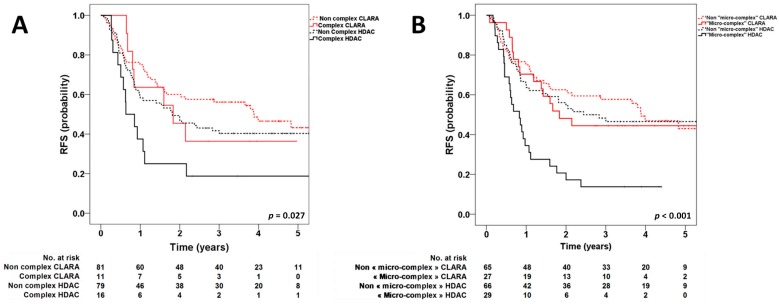
Kaplan–Meier estimates of relapse-free survival according to treatment arm (clofarabine-based consolidation (CLARA) vs. high-dose cytarabine (HDAC)) and complex karyotype (**A**) (4y-RFS: complex CLARA vs. non-complex CLARA, *p* = 0.49; complex HDAC vs. non-complex HDAC, *p* = 0.039; complex CLARA vs. complex HDAC, *p* = 0.13; non-complex CLARA vs. non-complex HDAC, *p* = 0.23) and micro-complex karyotype (**B**) (4y-RFS micro-complex CLARA vs. non-micro-complex CLARA, *p* = 0.58; micro-complex HDAC vs. non-micro-complex HDAC, *p* < 0.001; micro-complex CLARA vs. micro-complex HDAC, *p* = 0.004; non-micro-complex CLARA vs. non-micro-complex HDAC, *p* = 0.70).

Among patients with the complex karyotype, the CLARA-based consolidation was associated with a trend of a better RFS, although it did not reach statistical significance (4-year RFS (4y-RFS), 36.4% vs. 18.8%; *p* = 0.134) (Figure 1A). Interestingly, when considering all patients with the micro-complex karyotype, the CLARA consolidation significantly improved RFS (4y-RFS, 44.4% vs. 13.8%; *p* = 0.004) (Figure 1B). Similar results were observed when censoring at the time of allogeneic stem cell transplantation in first remission (*p* = 0.012). By contrast, the treatment arm did not impact RFS in patients without the micro-complex karyotype (4y-RFS, 46.9% vs. 46.6%; *p* = 0.698).

Considering the well-known association between *TP53* mutations and complex karyotypes among hematological malignancies [10], *TP53* sequencing was performed in the present cohort. A total of 15 patients (8%) carried at least one *TP53* mutation, among which 12 harbored a complex karyotype and 12 had a micro-complex karyotype. As expected, the *TP53* mutations statistically impaired RFS in the whole cohort (4y-RFS, 20.0% vs. 43.7%; *p* = 0.029).

In a multivariate analysis that took both *TP53* mutations, complex karyotypes, and micro-complex karyotypes into account, only micro-complex karyotypes remained a poor prognostic factor in the HDAC arm (HR, 2.32 (95% CI = 1.34–4.04); *p* = 0.003). By contrast, none of them had a prognostic value in the CLARA arm (Table 1). Only a few studies have assessed the prognostic value of SNP-array karyotyping in AML. A previous study by Bullinger et al. [4] showed that neither the presence nor the number of CNAs predict outcome, but this study focused on cytogenetically normal AML; this is in contrast to our cohort, which was enriched with patients who harbored abnormal cytogenetics. Another study by Parkin et al. [5] found that two or more SNP-array alterations negatively impacted overall survival, including when controlling for age and karyotype in multivariate analysis. A difference in the cut-off chosen by the authors may have been due to the use of different SNP-array chips, different patient selections, and different treatments. Overall, the effect observed in the present cohort may have reflected changes in mRNA expression at putative target genes that are involved in leukemogenesis and disease resistance. However, we did not identify any recurrent pattern of CNA or CN-LOH that was associated with a poor outcome. One could hypothesize that a high number of SNP-array alterations could reflect a genomic instability that is concordant with a more aggressive disease and resistance to cytarabine. Clofarabine added, either simultaneously with or in sequence, to cytarabine may overcome this factor by increasing cytarabine accumulation in AML cells [7].

## 4. Conclusions

In conclusion, our results demonstrate that SNP-array analysis could represent a powerful and reproductive tool to better detect patients with poor prognosis, especially in patients who lack a favorable risk factor in conventional genetics. Indeed, these results demonstrate that a SNP-array could refine the stratification of adult AML by identifying patients with a micro-complex karyotype (defined by at least four SNP-array abnormalities) who present a higher risk of relapse. Additionally, this study allowed us to characterize the relationship between the micro-complex karyotype and its prognostic significance in the context of alternative consolidation regimens. The current report enlarges our previous published findings about the benefits of the clofarabine-based consolidation in younger AML patients with intermediate or unfavorable cytogenetics. We suggest that this effect could be restricted to AML with micro-complex karyotypes, which represent an outstanding marker as compared to complex karyotypes or *TP53* mutations. However, broader prospective studies are required to further confirm the prognostic impact of micro-complex karyotypes.

## Figures and Tables

**Table 1:** Multivariate Cox model analysis of relapse-free survival (RFS).

Variables	Hazard Ratio	95%CI	*p*-Value
**HDAC**			
Micro-complex karyotype	2.32	1.34–4.04	<0.01
Complex karyotype	1.55	0.73–3.28	0.25
*TP53* mutation	0.77	0.3–1.99	0.59
**CLARA**			
Micro-complex karyotype	1.03	0.49–2.17	0.94
Complex karyotype	0.57	0.13–2.5	0.45
*TP53* mutation	4.83	0.92–25.25	0.06

## Supplementary Materials

The following are available online at https://www.mdpi.com/2072-6694/12/1/88/s1. Table S1: Patient characteristics at AML diagnosis.
