# Supplementary material for: Clofarabine Improves Relapse-Free Survival of Acute Myeloid Leukemia in Younger Adults with Micro-Complex Karyotype
Source: Cancers (Basel). 2019 Dec 30;12(1):88. doi: 10.3390/cancers12010088 (PMC7017244; doi:10.3390/cancers12010088)
Supplement: Supplementary file 1 [file cancers-12-00088-s001.pdf]

Supplementary Materials

Clofarabine Improves Relapse-Free Survival of Acute Myeloid Leukemia in Younger Adults with Micro-Complex Karyotype

Laurène Fenwart, Nicolas Duployez, Xavier Thomas, Nicolas Boissel, Sandrine Geffroy, Alice Marceau-Renaut, Denis Caillot, Emmanuel Raffoux, Emilie Lemasle, Jean-Pierre Marolleau, Céline Berthon, Meyling H. Cheok MSPharm, Pauline Peyrouze, Arnaud Pigneux, Norbert Vey, Karine Celli-Lebras, Christine Terré, Claude Preudhomme and Hervé Dombret

Table S1. Patient characteristics at AML diagnosis.

Characteristics	All (*n* = 187)	CLARA arm (*n* = 92)	HDAC arm (*n* = 95)	*p*-Value
**Median age, years**	48	50	45	0.07
**Sex (male/female), n**	101/86	48/44	53/42	0.66
**ELN risk classification**				0.41
Favorable, n (%)	1 (1)	0	1 (1)	
Intermediate, n (%)	137 (73)	69 (75)	68 (72)	
Unfavorable, n (%)	40 (21)	17 (18)	23 (24)	
Unknown, n (%)	9 (5)	6 (7)	3 (3)	
**ALFA risk classification**				0.66
Intermediate, n (%)	112 (60)	57 (62)	55 (58)	
Unfavorable, n (%)	75 (40)	35 (38)	40 (42)	
**Remission status**				0.59
CR/CRp in 1 course, n (%)	172 (92)	86 (93)	86 (91)	
Late CR/CRp, n (%)	15 (8)	6 (7)	9 (10)	
**ASCT in 1st CR, n (%)**	94 (50)	48 (52)	46 (48)	0.66
**Mutational status**				
NPM1, %	26	29	23	0.39
CEBPA, %	2	2	2	1.00
FLT3-ITD, %	29	30	28	0.74
TP53, %	9	6	11	0.28
**SNP alterations, median (range)**	2 (0–46)	1 (0–46)	2 (0–41)	0.41

ELN, European LeukemiaNet classification according to Döhner et al. (8); ALFA, Acute Leukemia French Association; CR, complete remission; ASCT, allogeneic stem cell transplantation.

© 2019 by the authors. Licensee MDPI, Basel, Switzerland. This article is an open access article distributed under the terms and conditions of the Creative Commons Attribution (CC BY) license (http://creativecommons.org/licenses/by/4.0/).
